# Properties of a Tightly Focused Circularly Polarized Anomalous Vortex Beam and Its Optical Forces on Trapped Nanoparticles

**DOI:** 10.1186/s11671-019-3089-5

**Published:** 2019-07-26

**Authors:** Yihua Bai, Miao Dong, Mingyan Zhang, Yuanjie Yang

**Affiliations:** School of Physics, University of Electronic Science and Technology of China, Chengdu, 610054 China

**Keywords:** Nanoparticles, Optical trapping, Circularly polarized anomalous vortex beam

## Abstract

The characteristics of a circularly polarized anomalous vortex beam (CPAVB), focused by an objective lens with a high numerical aperture (NA), are studied analytically and theoretically. It shows that the topological charge can affect the beam profile significantly and a flat-topped (FT) beam can be obtained by modulating the NA and topological charge. It is interesting to find that spin-to-orbital angular momentum conversion can occur in the longitudinal component after tight focusing. Furthermore, optical forces of the tightly focused CPAVB on nanoparticles are analyzed in detail. It can be expected to trap two kinds of nanoparticles using such beam near the focus.

## Introduction

Vortex beams with a spiral phase factor exp(*imθ*) have attracted extensive attention over the past two decades, where *m* is a topological charge and can be any integer value and *θ* is the azimuthal angle on a plane transverse to optical axis [1, 2]. Vortex beams have been widely used in numerous applications owing to their “doughnut” intensity profile and orbital angular momentum (OAM), such as optical tweezers [3–7], free-space optical communication [8], and quantum information [9]. Recently, researchers have paid more attention to the study of circularly polarized vortex beam because of its unique characteristics [10–15], for instance, it carries both spin angular momentum (SAM) and OAM at the same time. These unique characteristics can significantly expand and enhance the applications of vortex beams.

The tightly focusing characteristics of various beams under a lens system with high NA is another hot topic [16–20] for their important applications in particles trapping [21], microscopy [22], optical data storage [23], etc. Thus far, different beams have been studied, ranging from scalar vortex beams to vector vortex beams [10, 24–31]. For instance, Hao et al. [26] and Pu et al*.* [27] studied the properties of spirally polarized vortex beam under a high NA lens. It was shown that a flat-topped (FT) profile can be achieved and the OAM can be adjusted by choosing a proper polarized state in the focal plane. Zhan et al. studied the properties of tightly focused vortex beams with circularly polarization [10], showing that a strong longitudinal component can be produced.

Anomalous vortex beam (AVB), a novel beam which can evolve into elegant Laguerre-Gaussian beam in the far field, was proposed recently [32]. Such beam has attracted much attention and been widely investigated [33–38], owing to its extraordinary propagation properties. To the best of our knowledge, there is no report on the CPAVBs focused by a high NA lens. In this paper, the mathematical expressions of the CPAVBs after tight focusing are derived. Then we analyze the effect of beam order, topological charge, and NA value on the beam profile and phase distribution. At the last part, optical forces of tightly focused CPAVBs are studied.

## Methods

A circularly polarized beam can be written as follow, which indicates the linear superposition of radially and azimuthally polarized beams [10]:

1 $$ {\mathrm{E}}_{LHC(RHC)}=P(r){e}^{\pm i\varphi}\left({\mathrm{e}}_{\rho}\pm j{\mathrm{e}}_{\varphi}\right)/\sqrt{2} $$

where *P*(*r*) is the amplitude distribution. The sign “+” and “−” are left-hand and right-hand circular polarization, respectively. *e*_*ρ*_ and *e*_*φ*_ are the radial and azimuthal vectors in the cylindrical coordinates, respectively. And expressions of the radially and azimuthally polarized beam can be obtained in [39–41].

The scheme of the focusing system is the same as Ref. [42]. The pupil apodization function of AVB under a sine condition (i.e., *r* = *f*sin*θ*) can be written as [32, 38]:

2 $$ {\mathrm{E}}_{\mathrm{n},\mathrm{m}}\left(\theta, \varphi \right)={E}_0{\left(\frac{f\sin \theta }{w_0}\right)}^{2n+\left|m\right|}\exp \left(-\frac{f^2{\sin}^2\theta }{{w_0}^2}\right)\exp \left(- im\varphi \right) $$

where *f* is the focal length, *θ* varies from 0 to *α*, *α* is the maximal angle of NA, and *E*_0_ and *w*_0_ are a constant and waist radius, respectively. *n*, *φ*, and *m* are the beam order, azimuthal coordinates, and the topological charge, respectively.

According to the vector Debye theory, the expressions of the electrical field, of the tightly focused CPAVB in cylindrical coordinates, can be derived as Eq. (3):

3a $$ {\displaystyle \begin{array}{l}{E}_{\pm, \rho}\left(\rho, \varphi, z\right)=-\frac{ikf}{2}{\int}_0^{\alpha }{E}_0{\left(\frac{f\sin \theta }{w_0}\right)}^{2n+\left|m\right|}\exp \left(-\frac{f^2{\sin}^2\theta }{w_0}\right){i}^m\\ {}\kern6.399996em \times \sin \theta \sqrt{\cos \theta}\exp \left( ikz\cos \theta \right)\exp \left[i\left(m\pm 1\right)\varphi \right]\\ {}\kern6.399996em \times \left[\left(\cos \theta +1\right){J}_m\left( k\rho \sin \theta \right)-\left(\cos \theta -1\right){J}_{m\pm 2}\left( k\rho \sin \theta \right)\right] d\theta \end{array}} $$

3b $$ {\displaystyle \begin{array}{l}{E}_{\pm, \varphi}\left(\rho, \varphi, z\right)=-\frac{ikf}{2}{\int}_0^{\alpha }{E}_0{\left(\frac{f\sin \theta }{w_0}\right)}^{2n+\left|m\right|}\exp \left(-\frac{f^2{\sin}^2\theta }{w_0}\right){i}^{m\pm 1}\\ {}\kern6.399996em \times \sin \theta \sqrt{\cos \theta}\exp \left( ikz\cos \theta \right)\exp \left[i\left(m\pm 1\right)\varphi \right]\\ {}\kern6.399996em \times \left[\left(\cos \theta +1\right){J}_m\left( k\rho \sin \theta \right)-\left(\cos \theta -1\right){J}_{m\pm 2}\left( k\rho \sin \theta \right)\right] d\theta \end{array}} $$

3c $$ {\displaystyle \begin{array}{l}{E}_{\pm, z}\left(\rho, \varphi, z\right)=- ikf{\int}_0^{\alpha }{E}_0{\left(\frac{f\sin \theta }{w_0}\right)}^{2n+\left|m\right|}\exp \left(-\frac{f^2{\sin}^2\theta }{w_0}\right){i}^{m\pm 1}\\ {}\kern6.399996em \times {\sin}^2\theta \sqrt{\cos \theta}\exp \left( ikz\cos \theta \right)\exp \left[i\left(m\pm 1\right)\varphi \right]\\ {}\kern6.399996em \times {J}_{m\pm 1}\left( k\rho \sin \theta \right) d\theta \end{array}} $$

where *J*_*n*_(*α*) is a *n*-order Bessel function of the first kind and *k* = 2π/λ. We define *E*_+_ and *E*_−_ as the expression of the electrical field of right-hand and left-hand CPAVB, respectively.

In the above equations, the following formulas are used [43]:

4 $$ \left\{\begin{array}{l}{\int}_0^{2\pi}\cos \left( n\varphi \right)\exp \left[ ia\cos \left(\varphi -\phi \right)\right] d\varphi =2\pi {i}^n{J}_n(a)\cos \left( n\phi \right)\\ {}{\int}_0^{2\pi}\sin \left( n\varphi \right)\exp \left[ ia\cos \left(\varphi -\phi \right)\right] d\varphi =2\pi {i}^n{J}_n(a)\sin \left( n\phi \right)\end{array}\right. $$

Then, we can calculate the total intensity of the tightly focused CPAVB as follow:

5 $$ I={\left|{E}_{\rho}\left(\rho, \varphi, z\right)\right|}^2+{\left|{E}_{\varphi}\left(\rho, \varphi, z\right)\right|}^2+{\left|{E}_z\left(\rho, \varphi, z\right)\right|}^2 $$

where *E*_*ρ*_, *E*_*φ*_, and *E*_z_ are the amplitudes of corresponding components.

## Results and Discussion

### Tight-Focusing Characteristics of the CPAVB

**Fig. 1:**
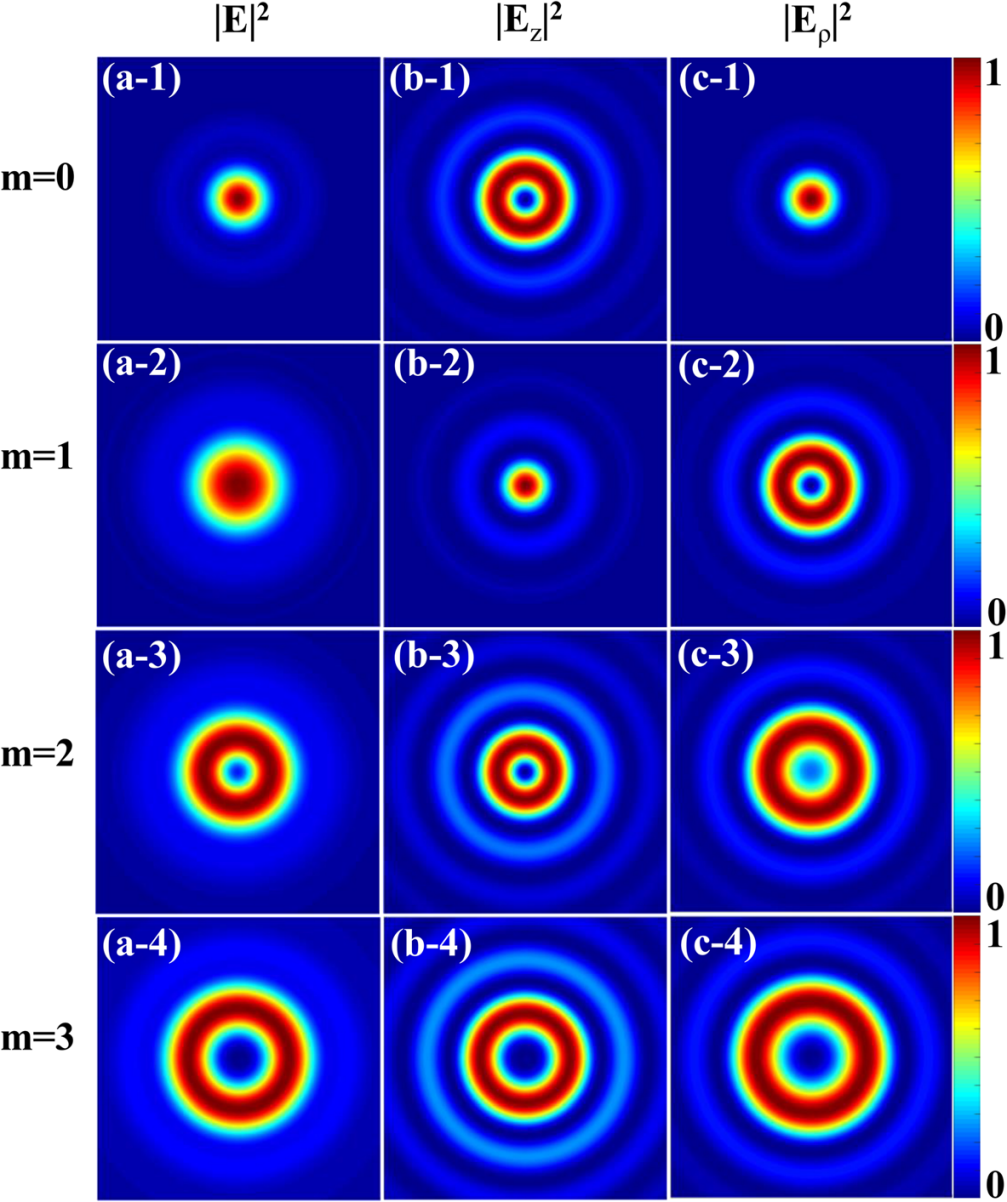
Intensity profile for the tightly focused left-hand CPAVBs with *n* = 1 for different topological charges. **a-1** to **a-4**, **b-1** to **b-4**, and **c-1** to **c-3** are the total intensity |*E*|^2^ and longitudinal |*E*_*z*_|^2^ and radial |*E*_*ρ*_|^2^ components, respectively

In this section, using the above equations, we study the properties of the tightly focused CPAVB. In the simulation, we set NA = 0.85, *λ* = 632.8 nm, *w*_0_ = 2 mm, and *f* = 2 mm. In Fig. 1, the total intensity profile and corresponding longitudinal and radial components of the left-hand CPAVBs with *n* = 1 for different topological charges in the focal plane are depicted, respectively. We can find that the total intensity is nonzero at the center when *m* ≤ 2, while there exists a dark spot in the center when *m* > 2. In addition, the radial component of focused fields is not zero on the axis when *m* = 0, 2, and the same as the longitudinal component when *m* = 1. These results can be explained from Eq. (3) and Eq. (5), owing to the fact that *J*_*m*_ always equals to zero at the origin except for *m* = 0. The Bessel function of the first kind in all three components is zero at the center when *m* > 2, and thus the total intensity is zero. Otherwise, there exists at least one component containing *J*_0_, which means the central intensity can be nonzero and maximum. What is more, for total and radial components, focal spot size increases as the topological charge increases. Therefore, we can conclude that the total intensity and focal spot size in the focal field are affected by topological charge.

**Fig. 2:**
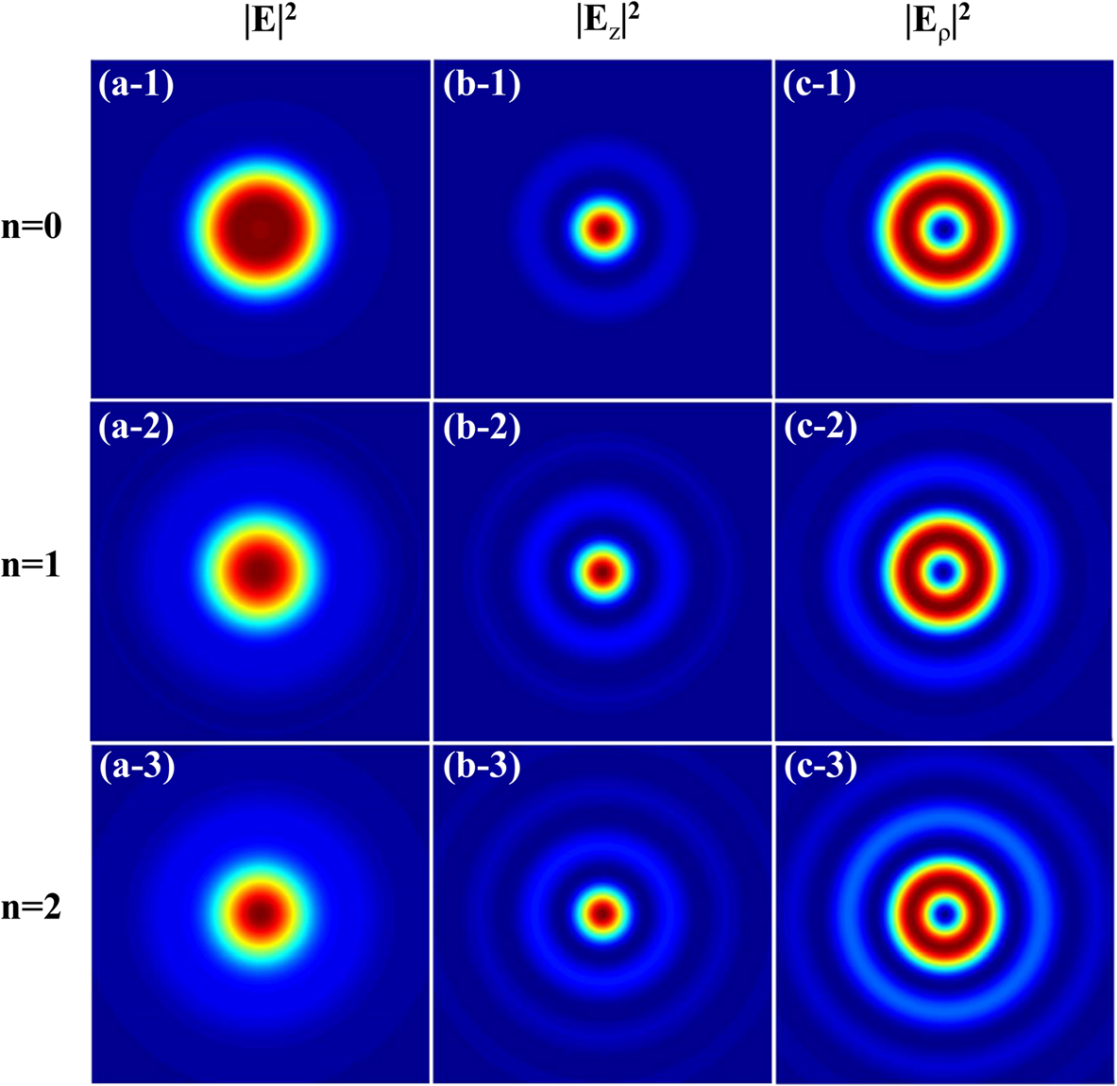
Intensity profile for the tightly focused left-hand CPAVBs with *m* = 1 for different beam orders. **a-1** to **a-3**, **b-1** to **b-3**, and **c-1** to **c-3** are the total intensity |*E*|^2^ and longitudinal |*E*_*z*_|^2^ and radial |*E*_*ρ*_|^2^ components, respectively

In Fig. 2, the total intensity profile and corresponding longitudinal and radial components of the left-hand CPAVBs with *m* = 1 for different beam orders in the focal plane are depicted, respectively. One can see that as *n* increases, the outer rings of each component and total intensity are gradually becoming brighter, while the pattern of the intensity does not change. Thus the beam order *n* does not affect the shape of the intensity patterns greatly.

**Fig. 3:**
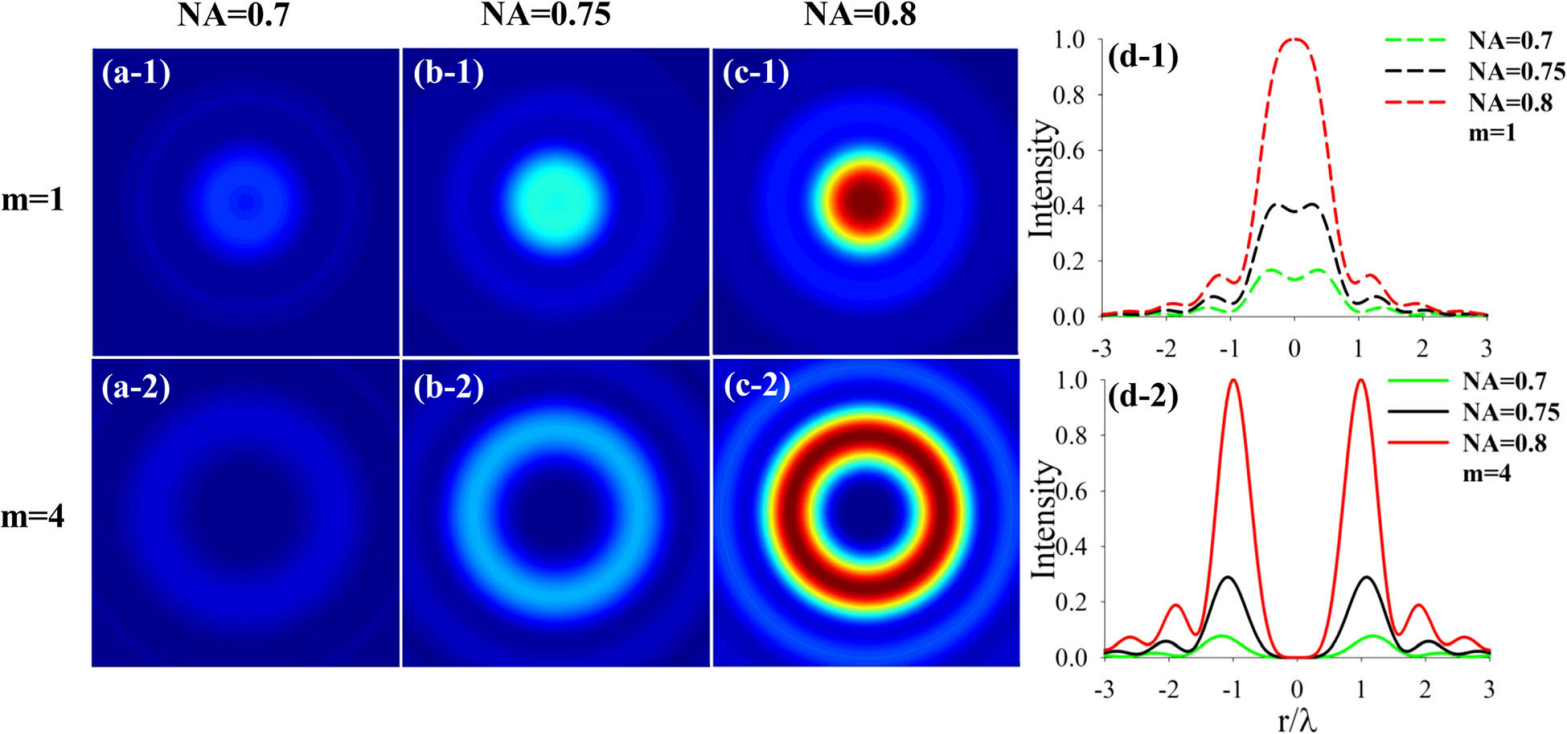
Variation of the intensity with the different NA of the left-hand CPAVBs with *m* = 1 and *m* = 4, respectively. **a-1** and **a-2**, **b-1** and **b-2**, and **c-1** and **c-2** are NA = 0.7, 0.75, 0.8, respectively. **d-1** and **d-2** Cross-section of the intensity

Then we study how the NA value influences the focusing properties of CPAVBs with *n* = 2 for *m* = 1 and *m* = 4, respectively. As shown in Fig. 3, it is noticeable that the central intensity remains nonzero for the case of topological charge *m* = 1, while central intensity is dark in the focal plane for *m* = 4. Comparing Fig. 3 d-1 with d-2, we can find that the intensity increases and gathers to the center with increasing NA. Especially, for the case of *m* = 1, a FT beam can be obtained when NA increases to 0.8.

**Fig. 4:**
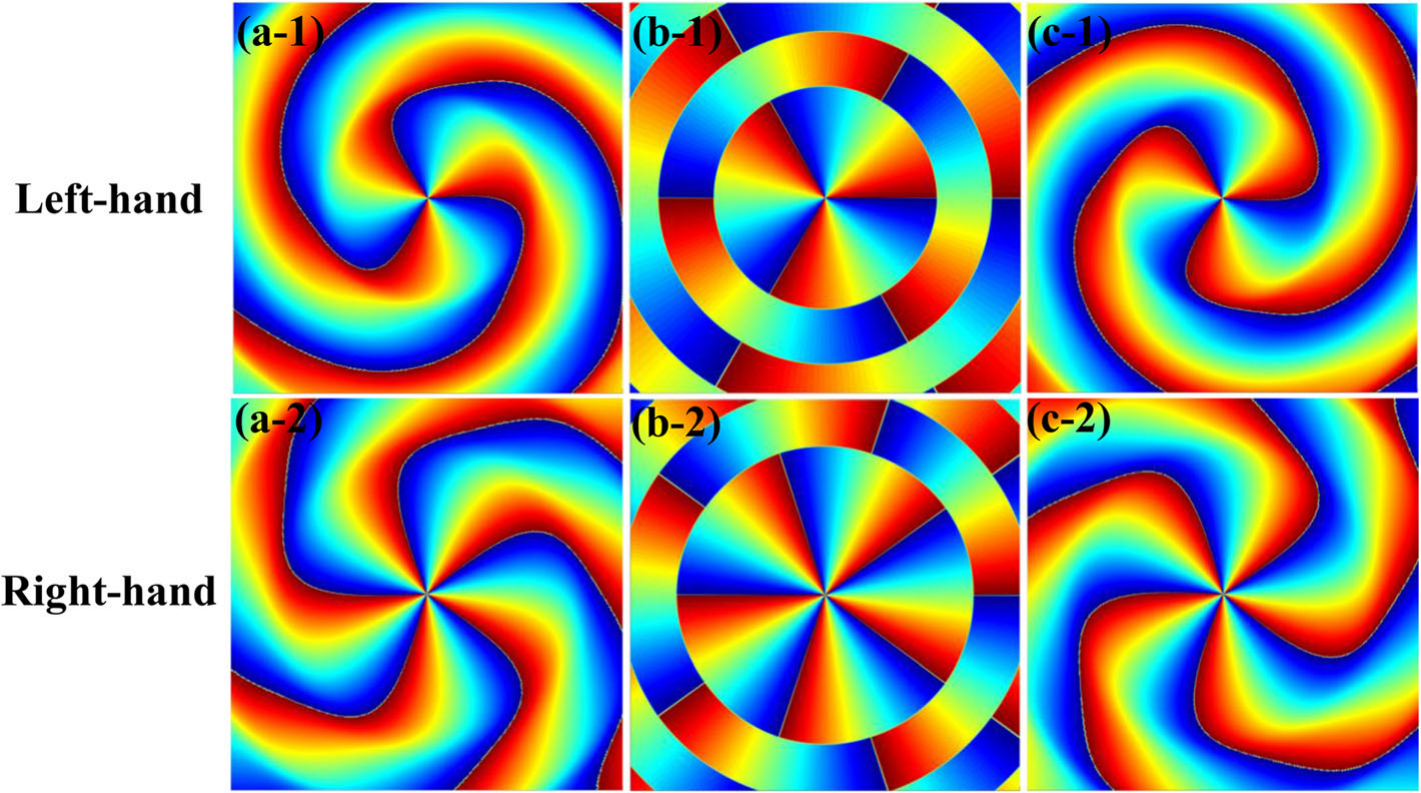
Phase profile of the longitudinal component of CPAVBs with *m* = 4 near the focus. The first and second rows are the left-hand and right-hand CPAVBs, respectively. **a-1** to **a-2** *z* = − 0.005*z*_*r*_. **b-1** to **b-2** *z* = 0. **c-1** to **c-2** *z* = 0.005*z*_*r*_

Based on Eq. (3c), we calculated the phase distributions of longitudinal component CPAVBs in the vicinity of focus, as shown in Fig. 4. The first and second rows of Fig. 4 are the left-hand and right-hand CPAVBs, respectively. The location for Fig. 4 a–c are *z* = − 0.005*z*_*r*_, 0, 0.005*z*_*r*_, respectively, where *z*_*r*_ = *kw*_0_^2^/2 is the Rayleigh range. Other parameters are set as *n* = 1 and NA = 0.85. As shown in Fig. 4, the contour of phase patterns changes from clockwise to anticlockwise after passing through the focal plane. Comparing Fig. 4 a-1 to c-1 with Fig. 4 a-2 to c-2, it is interesting to find that the topological charge near the focus changes from 3 to 5 when the left-hand CPAVB is replaced by a right-hand one. This phenomenon may be explained as a left-hand CPAVB with *m* = 4 carries SAM *l*_*s*_ = −*ħ* and OAM *m* = 4*ħ*. Owing to the compensation of the opposite OAM converted from SAM, the topological charges decrease to three after tight focusing. By analogy, we can expect the similar behavior of the right-hand CPAVB with *m* = 4, which carries SAM *l*_*s*_ = *ħ* and OAM *m* = 4*ħ*. Owing to OAM converted from SAM, the topological charges increase to five. Therefore, we can conclude that there is a conversion from SAM into OAM in the longitudinal component after tight focusing.

### Trapping Nanoparticles Using the Tightly Focused CPAVB

Based on the Rayleigh scattering theory [44], the scattering force and gradient force should be considered when discussing the optical trapping. The scattering force, written as ***F***_scat_ = ***e***_*z*_*n*_*m*_*αI*_out_/*c*, tends to destabilize the optical trap, where *c* is light velocity, ***e***_z_ is a unit vector along the *z* direction, *I*_out_ is the intensity of focused beam, *α* = (8/3)*π*(*ka*)^4^*a*^2^[(*η*^2^ − 1)^2^/(*η*^2^ + 2)^2^], *ɑ* is the nanoparticle’s radius, *η* = *n*_*p*_/*n*_*m*_, and *n*_*m*_ and *n*_*p*_ are refractive index of surrounding media and nanoparticle, respectively. And the gradient force (***F***_grad_) trends to pull a nanoparticle back to the focus, which can be expressed asF_grad_ = 2*πn*_*m*_*β* ∇ *I*_out_/*c*, where *β* = *a*^3^(*η*^2^ − 1)/(*η*^2^ + 2).

**Fig. 5:**
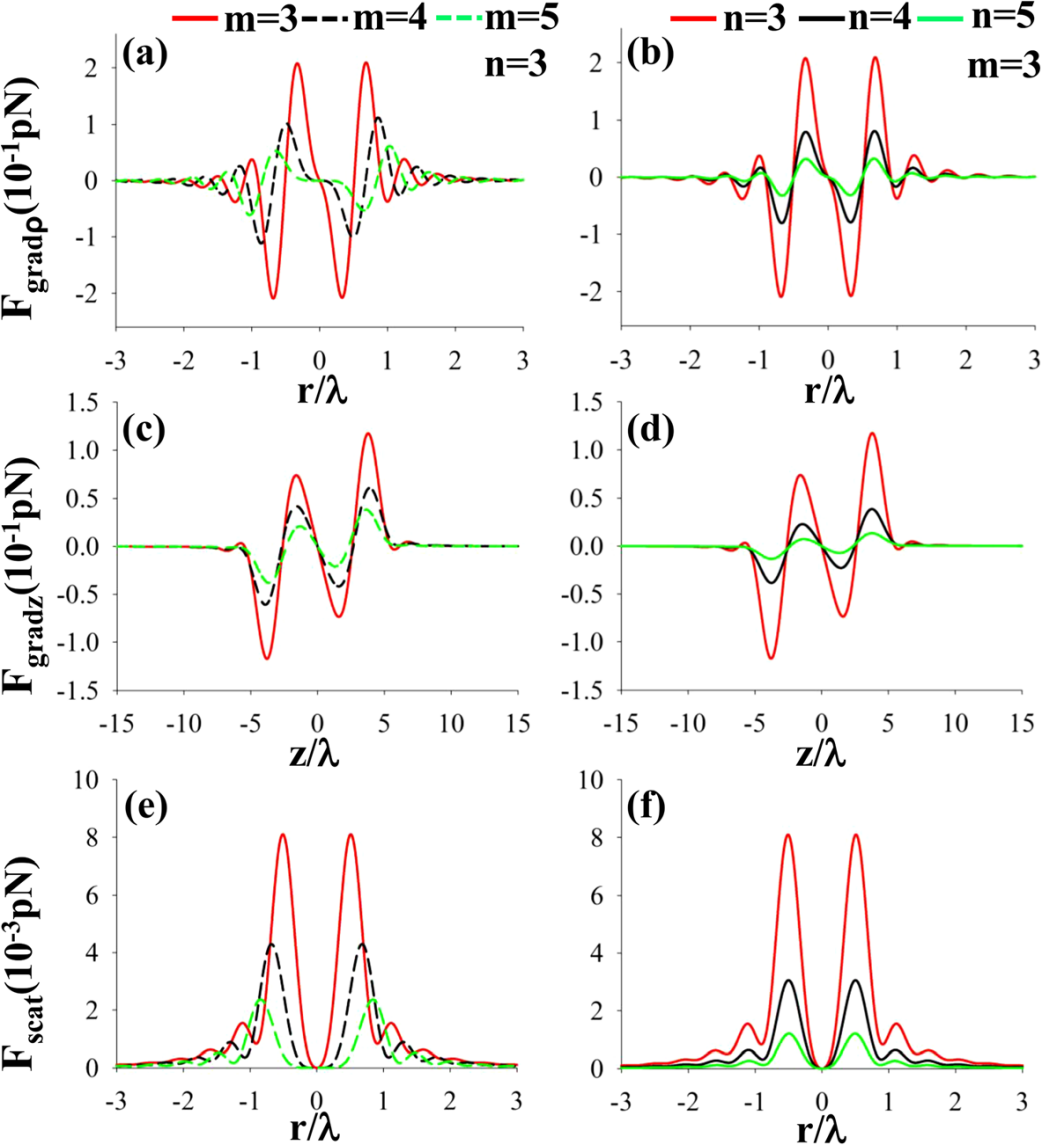
**a**–**f** The radial, longitudinal gradient forces and scattering forces of a left-hand CPAVB after tight focusing on a low refraction index particle *n*_*p*_ = 1

In the simulation experiment, we set *n*_*p*_ = 1.59 and *n*_*p*_ = 1 for glass and air bubble, respectively, *n*_*m*_ = 1.332, NA = 0.85, and *ɑ* = 50 nm. Figure 5 represents the radial, longitudinal gradient forces and scattering forces of a left-hand CPAVB on a nanoparticle with *n*_*p*_ = 1 for different *m* and *n*. The previous work shows that the total intensity is dark at the center when *m* ≥ 3. Therefore, as expected, for low refraction index nanoparticle, the radial and longitudinal gradient force will always pull the nanoparticle back to the focus, as shown in Fig. 5 a–d. Comparing with the gradient force, the scattering force is very small. Therefore, the low refraction index nanoparticle can be trapped stably.

**Fig. 6:**
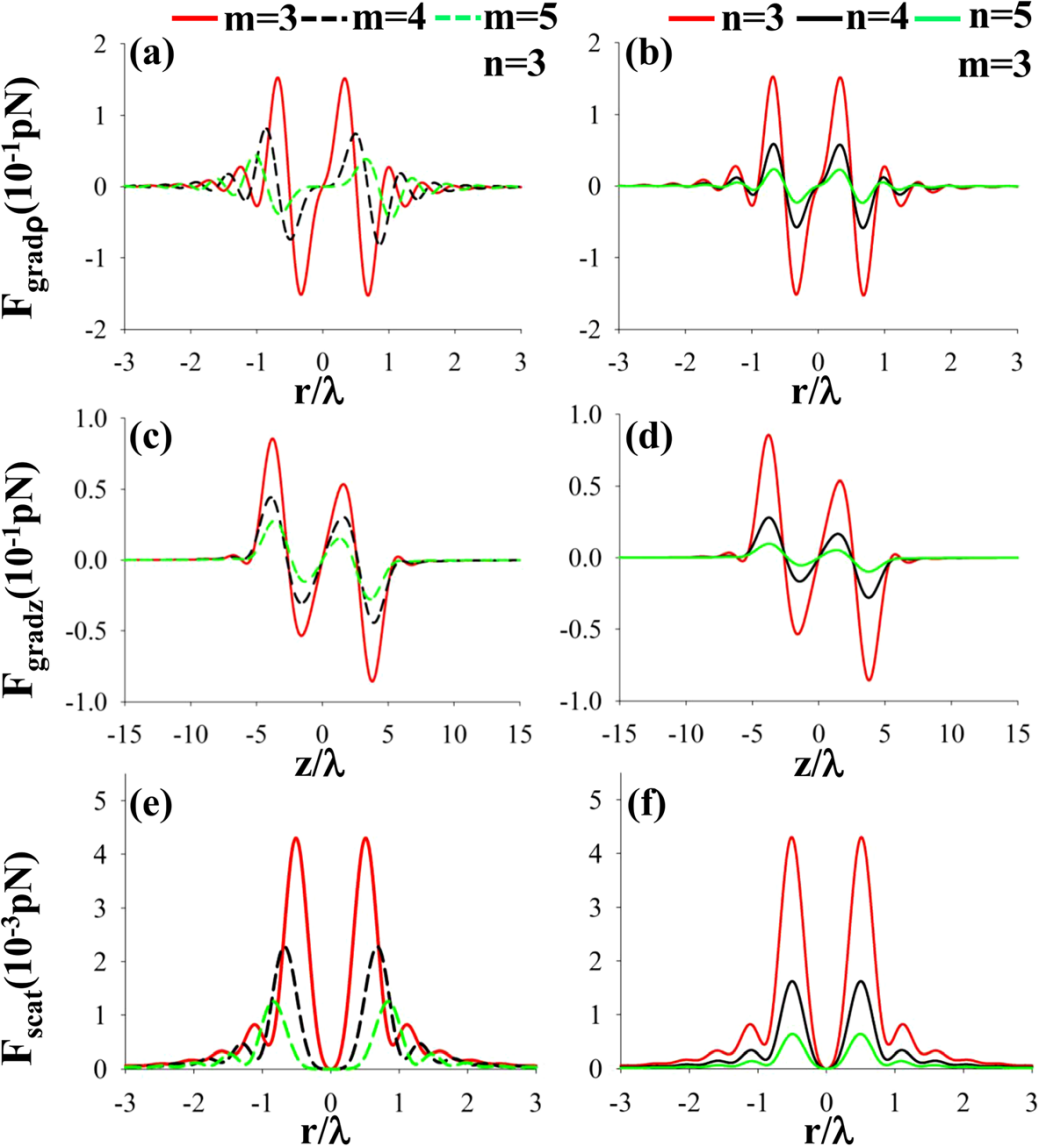
**a**–**f** The radial, longitudinal gradient forces and scattering forces of a left-hand CPAVB after tight focusing on a high refraction index particle *n*_*p*_ = 1.59

Figure 6 represents the radial, longitudinal gradient forces, and scattering forces of a left-hand CPAVB on a nanoparticle with *n*_*p*_ = 1.59 for different topological charges *m* and the beam orders *n*. From Fig. 6, we can see that there are several equilibrium points near the focus and the scattering force can be neglected compared with the gradient force. Therefore, the high refraction index nanoparticle can be captured near the focus.

## Conclusions

In this paper, the characteristics of tightly focused CPAVBs and their optical forces on nanoparticle have been discussed. We find that SAM of CPAVB can convert into OAM when such beam is tightly focused. Furthermore, tightly focused CPAVB can be used to trap two different kinds of nanoparticles, with low and high refraction index, near the focal plane. Our research will be of help for finding potential applications of CPAVB.

## Data Availability

The datasets generated and/or analyzed during the current study are available from the corresponding author on reasonable request.

## Abbreviations

AVB: Anomalous vortex beam
CPAVB: Circularly polarized anomalous vortex beam
FT: Flat-topped
NA: Numerical aperture
OAM: Orbital angular momentum
SAM: Spin angular momentum
